# A Narrative Review of Intestinal Microbiota’s Impact on Migraine with Psychopathologies

**DOI:** 10.3390/ijms25126655

**Published:** 2024-06-17

**Authors:** Miriam Francavilla, Sara Facchetti, Chiara Demartini, Anna Maria Zanaboni, Chiara Amoroso, Sara Bottiroli, Cristina Tassorelli, Rosaria Greco

**Affiliations:** 1 Department of Brain and Behavioral Sciences, University of Pavia, Via Bassi 21, 27100 Pavia, Italy; miriam.francavilla@mondino.it (M.F.); sara.facchetti@mondino.it (S.F.); annamaria.zanaboni@unipv.it (A.M.Z.); sara.bottiroli@mondino.it (S.B.); cristina.tassorelli@unipv.it (C.T.); 2 Headache Science and Neurorehabilitation Centre, IRCCS Mondino Foundation, Via Mondino 2, 27100 Pavia, Italy; chiara.demartini@mondino.it; 3 Gastroenterology and Endoscopy Unit, Fondazione IRCCS Cà Granda, Ospedale Maggiore Policlinico, 20135 Milan, Italy; chiara.amoroso@policlinico.mi.it

**Keywords:** microbiota, migraine, depression, anxiety, gut–brain axis

## Abstract

Migraine is a common and debilitating neurological disorder characterized by the recurrent attack of pulsating headaches typically localized on one side of the head associated with other disabling symptoms, such as nausea, increased sensitivity to light, sound and smell and mood changes. Various clinical factors, including the excessive use of migraine medication, inadequate acute treatment and stressful events, can contribute to the worsening of the condition, which may evolve to chronic migraine, that is, a headache present on >15 days/month for at least 3 months. Chronic migraine is frequently associated with various comorbidities, including anxiety and mood disorders, particularly depression, which complicate the prognosis, response to treatment and overall clinical outcomes. Emerging research indicates a connection between alterations in the composition of the gut microbiota and mental health conditions, particularly anxiety and depression, which are considered disorders of the gut–brain axis. This underscores the potential of modulating the gut microbiota as a new avenue for managing these conditions. In this context, it is interesting to investigate whether migraine, particularly in its chronic form, exhibits a dysbiosis profile similar to that observed in individuals with anxiety and depression. This could pave the way for interventions aimed at modulating the gut microbiota for treating difficult-to-manage migraines.

## 1. Introduction

Migraine is a highly prevalent and disabling neurological disorder characterized by recurrent episodes of unilateral, throbbing, moderate to severe headaches lasting 4 to 72 h with typical accompanying symptoms such as nausea, hypersensitivity to environmental stimuli (e.g., photophobia and phonophobia) and mood changes [1]. According to the latest edition of the Global Burden Disease study, migraine is the second leading cause of disability worldwide, provoking a great socio-economic burden and a significant impact on healthcare [2]. Migraine has a prevalence of 24% in the general population, with a variation related to gender difference, affecting women disproportionately compared to man at a rate of 3:1 [2].

The International Headache Society classifies migraine into several clinical subtypes, depending on frequency and whether it is accompanied by a migraine aura [3,4]. Aura consists in sensory or motor manifestations (positive or negative) that usually occur before a migraine episode. Depending on the headache frequency, migraine can be classified as episodic (EM) or chronic migraine (CM). In the former, the patients experience less than 15 headache days/month, while CM is defined as 15 or more headaches per month, of which 8 retain migrainous characteristics, for more than 3 months [3,4]. Of note, the conversion to CM has been estimated to occur in 3% of EM patients per year. The chronification process and the occurring neurological changes are influenced by several clinical risk factors, like the overuse of acute migraine medication, ineffective acute treatment and stressful events [5,6,7]. CM is associated with a higher disability and a wide range of comorbidities that can further complicate the prognosis, treatment and clinical outcome [7,8,9]. Among the numerous disorders that can occur as comorbidities with migraine, anxiety and mood disorders, especially depression, are the most common [10]. Patients with migraine are 2.5 times more likely to develop a depressive disorder [11]. Indeed, subjects with migraine have a higher prevalence of psychopathological comorbidities than the general population, and the risk of a first-ever migraine is three times higher in subjects with a depressive disorder than in those without it [11]. Depressive disorders, especially major depressive disorder (MDD), are characterized by a persistent depressed mood and/or a loss of interest/pleasure, lasting at least two weeks. Almost two-thirds of individuals with MDD have a manifestation complicated by anxiety [12,13,14]. Like for depression, anxiety disorders are much more prevalent among people with migraine, especially those with CM, than they are in the general population [15]. Numerous clinical and preclinical research works demonstrated the role of physiological processes driving inflammatory and stress responses in the development of depression and anxiety [16], as well as in the pathogenesis of migraine [17]. High levels of stress originating from stressful life events, daily difficulties or other sources have been linked to an increased risk of new-onset migraine and depression [18,19]. Indeed, chronic stress is recognized as a risk factor for migraine and depression, and the interaction between depression and migraine can largely be explained as stressors that increase the risk of each other [20]. In this scenario, the complex pathogenic process of either migraine and depression/anxiety is likely to involve biopsychosocial features and neurotransmitters dysregulation associated with stress [21]. Particularly, a serotonin (5-HT) imbalance may contribute to the pathogenic process of both MDD and migraine [22]. Psychopathologies may influence the progression of migraine to CM [23], and recent studies showed that personality/psychological disorders are components of migraine itself, which may influence the response to prophylactic treatment [24]. Similarly, psychological vulnerabilities were shown to negatively affect the outcome of the detoxification program in migraine patients who overuse acute medication for headaches [25,26]. The abovementioned findings imply that people with migraine bearing psychological comorbidities display a difficult-to-treat migraine [23].

Growing research has shown that changes in the composition of the gut microbiota are deeply involved in the pathophysiology of many diseases including neuropsychiatric (e.g., anxiety and depression) and neurological diseases, like migraine. Indeed, gut microbiota can alter the bidirectional communication network between the gastrointestinal (GI) tract and the central nervous system (CNS), known as the gut–brain axis (GBA) [27]. The manipulation of the gut microbiota has been used to treat these disorders [28], thus supporting a key role of the gut microbiota–GBA. In this regard, it is crucial to understand whether possible comorbidities share with migraine similar microbiota dysbiosis and whether specific approaches can represent a valid and innovative adjuvant therapy for the treatment of complicated migraine [29]. The GBA process is influenced by neurological, hormonal and humoral factors, which may contribute to the development of migraine and psychopathologies, suggesting that microbiota dysbiosis may involve overlapping biological mechanisms in both disorders [27,29].

In this narrative review, we summarize the available evidence on the microbiota composition in subjects with migraine and depression/anxiety disorders with the aim of providing insights into a possible common role of the microbiota–GBA in the two pathologies. In the following sections, we will first provide a general description of the GBA and then an overview of the main clinical and preclinical observations about the microbial profile in migraine and anxiety/depressive disorders. Afterward, we will provide a detailed description of the bacterial species and linked mechanisms that are shared by the two pathologies to gather insights into the potential involvement of the altered microbiota in the drug response of migraine comorbid with psychological disorders. 

## 2. Material and Methods 

A literature search was conducted on the PubMed database from 1998 until 2024 to address the review objective. The search string used for the research was: [migraine and microbiota] or [depression or anxiety and microbiota] or [animal model of migraine and microbiota] or [psychological disorders and microbiota] or [animal models, depression and microbiota]. The research methodology included microbiota studies conducted in patients with migraine and depression/anxiety. We also included preclinical studies involving animal models (rats or mice) reproducing one or more pathophysiological features of migraine pain and the most associated psychological diseases. Studies involving the pediatric population were not considered.

## 3. The Microbiota–Gut–Brain Axis

The GBA network consists in the afferent and efferent neurons transmitting signals via the autonomic nervous system to the enteric nervous system and the hypothalamic-pituitary–adrenal (HPA) axis, which are interconnected and controlled by many neurohumoral variables [27]. These connections allow signals from the gut to affect behavior by traveling through the vagus nerve to the CNS. At the same time, the CNS can influence gut functions, including GI motility, intestinal permeability and mucosal immune responses, throughout the sympathetic and parasympathetic branches of the autonomic nervous system and by releasing neuroendocrine peptides [30]. 

It has been recently recognized that gut microbiota play a major role in modulating the GBA, leading to a shift in the understanding of the traditional GBA toward the microbiome–GBA interaction [31]. Among the microorganisms colonizing the human GI tract, the *Firmicutes* and *Bacteroidetes* phyla account for 90% of the gut microbiota [32]. They establish a symbiotic connection with the host and affect numerous physiological processes, such as protection against pathogens, interaction with the immune system and metabolic functions [33,34]. However, the gut microbiota composition changes continuously throughout life due to environmental factors such as diet, lifestyle, infections and antibiotic therapy, contributing to several neurological disorders’ pathophysiology [35]. Stress related to psychological and physical factors or pharmacological treatment appears to be involved in microbiota dysbiosis in both diseases, leading to the activation of the HPA axis. Changes in the microbiota may influence levels of certain neurotransmitters, particularly 5-HT, as well as the peripheral inflammatory state and its reflection in the CNS. 

Current evidence indicates that the bottom-up modulation of the CNS by gut microbiota occurs through microbe-derived metabolites, such as short-chain fatty acids (SCFAs), secondary bile acids and tryptophan metabolites [36]. At the gut level, these metabolites interact with receptors on enteroendocrine and enterochromaffin cells, stimulating the secretion of 5-HT [37] and hormones [38]. Specifically, SCFAs can cross the intestinal barrier, enter systemic circulation and then cross the blood–brain barrier (BBB) via monocarboxylate transporters located on endothelial cells, influencing BBB integrity by modulating the expression of tight junction proteins [39]. SCFAs can also serve as signaling molecules and protect the BBB by binding to G protein-coupled receptors and free fatty acid receptors on brain endothelial cells [40]. Although the precise mechanisms involved in the action of SCFAs on the central nervous system remain largely unknown, several studies suggest their influence on neurological and behavioural processes [41].

Beside the abovementioned metabolites, several strains of gut bacteria can synthesize and release neurotransmitters, such as γ-aminobutyric acid (GABA), 5-HT, dopamine (DA) [42] and glutamate, all of which are involved in the pathophysiological mechanisms of migraine and depression. Another route of communication between gut microbiota and CNS is the immune system. Indeed, gut microbiota interact with local immune cells, shaping immune homeostasis and local immune responses toward a pro-inflammatory or anti-inflammatory state [43]. Intestinal immune cells can identify invasive pathogens and prevent their passage from the intestinal lumen into the circulation [44]. Under dysbiosis conditions, harmful bacteria stimulate the production of inflammatory cytokines by immune cells, leading to a sustained inflammatory state and the disruption of the intestinal barrier through the reduced expression of tight junction proteins [45]. This effect leads to the translocation of bacteria and bacterial toxins, such as lipopolysaccharide (LPS), into the systemic circulation, triggering low-grade inflammation [46]. 

## 4. Gut Microbiota Composition in Migraine: Clinical and Preclinical Data

A growing amount of evidence has found dysbiosis of the gut microbiota in subjects with migraine [47,48,49]. Moreover, the connection of gut microbiota and GBA is involved in the development and progression of migraine. Individuals with frequent migraine attacks also often experience GI symptoms such as reflux, diarrhea and constipation [50]. A whole metagenome association study found that alpha diversity evidently decreased in people with migraine compared to healthy subjects. This was associated with the lack of many beneficial bacteria, such as *Faecalibacterium prausnitzii* and *Bifidobacterium adolescentis* [47]. Moreover, it was reported that individuals with migraine had considerably higher levels of unfavorable bacterial characteristics, such as *Firmicutes*, particularly *Clostridium* spp., but also *Egghertella lenta* and *Ruminococcus gnavus* [47]. He et al. [29] found that *Actinobacteria* and *Catenibacterium*, are risk factors for migraine, while *Butyricicoccus* seems to be a protective factor for migraine and *Bifidobacterium* may decrease the risk of migraine [29]. Accordingly, in a randomized double-blind controlled study, probiotic supplementation with *Bifidobacterium* was able to improve migraine symptoms [51]. 

In a recent study comparing migraine patients and healthy individuals, Kopchak et al. [52] found significant differences in the quantitative composition of some gut resident microorganisms: *Alcaligenes* spp., *Clostridium coccoides*, *Clostridium propionicum*, *Eggerthella lenta*, *Pseudonocardia* spp. and *Rhodococcus* spp., and microscopic fungi *Candida* spp. and *Micromycetes* spp., were more abundant in CM and EM patients than in the healthy controls. Additionally, a negative correlation was found between elevated levels of *Clostridium coccoides* and the MIDAS score (Migraine Disability Assessment Test), while a positive correlation was detected between the *Eggerthella lenta* level and the VAS score (Visual Analog Scale, used for assessing headache pain) [52]. Another study reported that *Tissierellia* and *Peptoniphilaceae* were more abundant in CM and EM at the class, order and family levels when compared to controls [49]. All patients had higher genus abundances of *Roseburia*, *Eubacterium_g4*, *Agathobacter*, *PAC000195_g* and *Catenibacterium* [49]. The same study also reported that *Catenibacterium* was significantly altered according to the presence of anxiety in the EM and CM groups compared to the control group. A significant association between microbiota composition changes and the clinical characteristics of migraine was found: a higher abundance of *PAC000195_g* was significantly associated with a lower headache frequency, and *Agathobacter* had a negative association with severe headache intensity [49]. In addition, the *Prevotellaceae* family, specifically *Prevotella7*, increases the risk of migraine in a two-sample Mendelian randomization study [53]. In another study, high levels of *Prevotella* and *Veillonella* and lower levels of *Rothia*, bacteria contributing to nitric oxide homeostasis, were observed in the saliva of individuals with migraine compared to controls [54]. Taking into consideration that nitrogen compounds have been identified as a migraine trigger and nitric oxide is involved in nociceptive transmission, a potential connection between migraine and these bacteria can be further suggested.

The availability of reliable animal models that mimic some of the key features of the diseases allowed the researchers to additionally investigate the underlying pathophysiological mechanisms. For the study of migraine, one of the most used and validated animal models is the one based on the systemic administration of nitroglycerin (NTG). This model can mimic both acute and chronic features of the disease, such as allodynia, hyperalgesia and the associated symptoms like anxiety and depression [55,56]. This model was indeed the most used in this field to study the alteration of the microbiota, yielding insights into the underlying mechanisms, such as abundance and diversity in a given pathology. In this model, as well as in another model of migraine pain, based on the induction of dural inflammation, differences in the gut microbial composition were reported between migraine-like animals and controls [57,58]. Although the findings were obviously not in complete agreement with clinical studies, a link between microbiota/metabolic variations and the pathogenesis of migraine is confirmed. Specifically, a reduction in *Bacteroidetes* and *Muribaculum* spp. concentrations and an increase in *Alistipes* were found in the NTG group compared with the control group [59]. SCFA treatment attenuated NTG-induced hyperalgesia, decreased the expression of pro-inflammatory mediators [60] and increased species belonging to the *Lactobacillus* genus [59]. This latter was found to be particularly altered in the dural inflammation model as well [58], in which the abundance in *Lactobacilli* was restored by topiramate treatment. *Lactobacillus* spp. is a well-studied and crucial bacterial genus that is a basal component of the intestinal microbiota and plays a role in several beneficial functions. The administration of different strains of *Lactobacillus* were shown to improve the absorption of SCFA by GI epithelial cells [61]. Treatment with probiotics containing different bacterial strains, including several *Lactobacillus* species, could relieve or reduce the duration and frequency of migraine attacks, thus improving the quality of life [62]. 

Changes in microbiota metabolites were also reported in the chronic NTG model, together with a significant reduction in the thermal threshold. In that model, an alteration in 30 bacterial species was found, with a significant decrease in thick-walled bacteria and an increase in the relative abundance of *Bacteroides* [63]. Among these altered bacteria, the NTG group had an increase in the relative abundance of *Prevotella*_1, *Ruminococcus*_2, *Streptococcus* and *Escherichia-Shigella* and a decrease in the relative abundance of *Desulfovibrio* and *Coprococcus*_1 [63]. Interestingly, *Prevotella_1* is elevated in women with anxiety and migraine [64], and a decrease in *Coprococcus* bacteria was also found in patients with migraine and depression [65]. Another study revealed differences in the gut microbiota between a chronic NTG mouse model and a vehicle group. Specifically, *Akkermansiaceae* was the most abundant taxon in control mice, and *Lachnospiraceae* was the most abundant group in NTG-treated mice, highlighting their potential role in migraine [66]. *Akkermansia muciniphila* was associated with improved host metabolic function, immune response and intestinal homeostasis; also, a positive correlation between its decrement and specific metabolites indicates potential protective action against migraine [67].

In the dural inflammation model, Nann et al. [68] highlighted that *Rhodococcus* was related to 5-HT synthesis, while *Adlercreutzia* and *Prevotella* showed a substantial correlation with plasma 5-HT levels. Additionally, animals displayed behaviors similar to depression and anxiety, both related to a deficiency in 5-HT in the CA1 and CA2 regions of the hippocampus [69]. The induction of dural inflammation led to changes in the metabolic pathways of the gut microbiota, resulting in elevated levels of butyrate, propionate and tryptophan, as indicated by the production of fecal metabolites such as indole-3-acetamide [58]. Other studies suggested that the gut microbiota might be involved in normal mechanical pain sensation, and migraine pain and dysbiosis could be critical in the chronicity of migraine [70]. Moreover, Kang et al. [71] demonstrated increased basal sensitivity and the upregulated expression of trigeminal Tumor necrosis factor alpha (TNFα) in germ-free mice or antibiotic-treated mice compared to pathogen-specific free mice. 

The abovementioned clinical and preclinical data support the interplay occurring between gut microbiota and migraine pathophysiology and associated comorbidities. In Table 1, the main altered microbial species observed in either migraineurs or animal models are summarized, together with the relevant effects that were observed.

## 5. Gut Microbiota Composition in Depression and Anxiety: Clinical and Preclinical Data

Mood disorders and anxiety are major clinical concerns and leading causes of disability and death worldwide [72]. Some pathophysiological mechanisms underlying the development of these psychopathologies have been hypothesized, including the involvement of immunological, neurotransmitter and hormonal pathways. In recent years, growing evidence has found a link between dysbiosis and psychological conditions, particularly in terms of microbial diversity and the relative abundance of specific bacterial taxa [73,74]. Because of the huge variety and complexity of psychopathological disorders, a great heterogeneity in terms of microbiota diversity is reported among studies [75,76]. In depression and anxiety, at the genus level, a decreased abundance of *Faecalibacterium*, *Roseburia* [77,78,79,80] and *Ruminococcus* was reported, specifically *Ruminococcus gnavus* [78,80]. In contrast, another study has shown a positive correlation between *Ruminococcus gnavus* and the DASS depression score (Depression, Anxiety and Stress Scale) [81]. Discrepancies were observed at the family level, with fluctuations in the abundance of *Lachnospiraceae* and *Ruminococcaceae* across available research. In depressed patients, *Firmicutes*, *Actinobacteria* and *Bacteroidetes* are the most affected bacteria at the phylum level, compared to healthy controls; specifically, MDD patients showed an increase in *Bacteroidetes* and *Actinobacteria*, while *Firmicutes* were decreased [77]. Additionally, in depressed patients, an enrichment of the genera *Bacteroides* [82] and *Eggerthella* [83] and a depletion of *Sutterella*, *Blautia*, *Faecalibacterium* and *Coprococcus* were reported [78,79,84]. Furthermore, an increase in the relative abundance of proinflammatory bacteria, such as the genera *Desulfovibrio*, *Eggerthella* and *Alistipes* was found in the MDD patients compared to the healthy controls [78,85,86]. Recent studies reported similar results in depression and anxiety; for instance, patients with anxiety show taxa involved also in depression, including reduced levels of *Prevotellaceae* and, subsequently in the *Prevotella* genus, *Faecalibacterium*, *Coprococcus* and *Dialister*, alongside elevated levels of *Enterobacteriaceae* [79,83,87], which suggests that, depending on the co-existence of both disorders, some of them may increase or decrease. 

Other studies have reported a reduction in the genera *Coprococcus*, *Faecalibacterium* and *Ruminococcus* [78,79,84,87], producing SCFAs, primarily butyrate and propionate metabolites [88,89]. Sanada et al. [90] further confirmed a reduction in *Coprococcus* and *Bifidobacterium*, along with diminished total SCFAs, in fecal samples of MDD patients compared to controls [91,92]. In several studies, MDD was characterized by an increased abundance of *Clostridium*, specifically *Clostridium propionicum* [78,93], as well as significantly higher levels of *Catenibacterium* [94]. Naseribafrouei et al. [86] demonstrated an increase in *Alistipes* in cases of depression, especially when associated with fatigue and stress. This increase contributed to an alteration in indole levels, affecting the GBA and reducing the availability of 5-HT.

In addition, certain strains of gut bacteria may synthesize and release neurotransmitter-like molecules, potentially contributing to MDD by modulating their levels [95]. Among these, glutamate, produced by *Lactobacillus*, appears to be implicated in anxiety-like behavior [96], while *Bifidobacterium* is known to produce both GABA and acetylcholine [97].

Preclinical models demonstrated that rodents with a gut microbiota alteration exhibit anxious and depressive behaviors normalized by bacterial probiotics administration [98,99]. Additionally, fecal transplantation from depressed patients into microbiota-depleted rats induced a depressive phenotype related to gut microbiota richness, diversity, tryptophan metabolism and immune function [100]. *Bifidobacterium* and *Lactobacillus* are the main genera showing beneficial effects on anxiety and depression-like behavior [98]. Among the *Lactobacillus*, *Lactobacillus Rhamnosus* reduces anxiety and depression-related behavior in experimental models [101]. Like *Lactobacillus*, the administration of *Bifidobacterium Longum* restored hippocampus BDNF levels and reduced anxiety-like behavior induced by inflammation [102,103], while no difference in serum BDNF was found in patients undergoing probiotic treatment [104]. Additionally, chronic treatment with *Bifidobacterium Infantes* alleviated stress-induced immune changes in early life and depression from maternal separation [105]. 

According to the monoamine deficiency hypothesis in depression [106], Cheng et al. [107] showed that *Akkermansia muciniphila* directly influences the host 5-HT system and can increase 5-HT levels in the host gut. A reduction in the genera *Akkermansia* and *Ruminococcus* was also evidenced by Ma et al. [108] in a model of chronic paradoxical sleep deprivation-induced depression in rats. In line with this, McGaughey et al. [109] found that *Akkermansia muciniphila* is negatively correlated with depression in mice models of social defeat. In agreement, *Akkermansia muciniphila* treatment significantly ameliorated depressive-like behavior in mice and restored abnormal variations in depression-related molecules (corticosterone, dopamine and BDNF) [110]. By contrast, *Faecalibacterium*, specifically *Faecalibacterium prausnitzii*, has been shown to have anxiolytic and antidepressant-like effects and to reverse the impact of chronic unpredictable mild stress in rats [111]. 

Table 2 summarizes the main altered microbial species in depression/anxiety disorders and related animal models, together with the relevant effects that were observed.

## 6. Migraine and Psychological Disorders Share an Altered Gut Microbiota Composition

**Figure 1:**
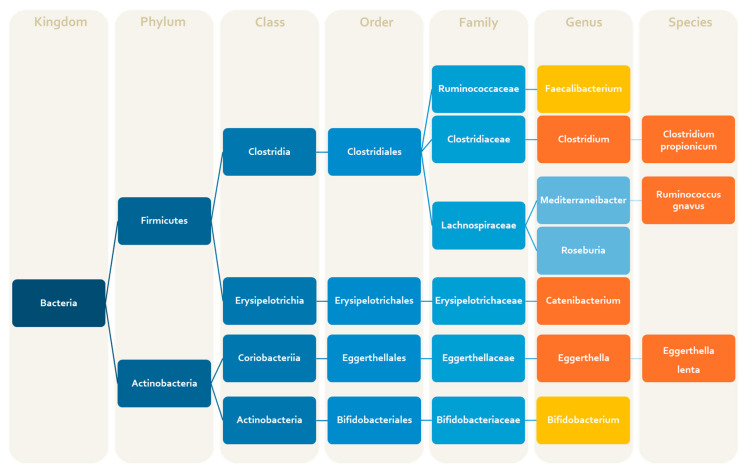
Visual representation of the common changes in the microbiota composition in subjects with migraine or depressive/anxiety disorders, highlighting the taxonomic classification of dysbiotic bacteria. The orange box stands for an increase and the yellow one stands for a decrease.

Neuropeptides, sex hormones, glutamate/GABA and the immune system are involved in the pathophysiological mechanisms of both migraine and depression/anxiety. Consequently, it is plausible that changes in the microbial population might also trigger depression in these subjects, especially since antimigraine drugs can themselves alter the gut flora [113]. Preclinical and clinical research linked changes in the gut microbiome to mental health problems, such as depression and anxiety, [65,114,115], and microbiota dysbiosis also appears to represent an important trigger for the onset of migraine and for its chronic development [116]. However, few studies have investigated microbiota changes associated with depression and anxiety in people with migraine [49,52]. Despite the limited number of studies, there is evidence of a decrease in *Faecalibacterium* [47,79] and *Bifidobacterium* [29,91] in migraine and psychiatric disorders. *Catenibacterium* [29,94], as well as *Clostridium propionicum* [52,93] and *Ruminococcus gnavus* [47,81], were found to be overrepresented (Figure 1). These microbial signatures shared by migraine and depressive disorders could become a valuable tool for detecting abnormal conditions in microbiomes in migraine with depressive comorbidities which are often resistant to drug treatment. 

The lipid metabolic function of the gut microbiota can affect the production of SCFAs and further affect host systemic inflammation [117]. In addition, high lipid metabolism and its end products may help to maintain the integrity of the gut barrier and influence the therapeutic effect of probiotics [118]. 

*Bifidobacterium* and *Faecalibacterium* produce SCFAs and have a large capacity to metabolize carbohydrates; specifically, mono- and disaccharides and their derivative bioactive metabolites (such as SCFA) are involved in microbiota–host chemical communication [119,120,121,122] and play a role in the integrity of the gut barrier [123]. In addition to influencing gut immunity, SCFAs can enter the CNS via the circulation and exert neuroprotective and anti-inflammatory effects [123]. Of interest, the gut microbiota of the healthy controls were more active in energy metabolism and SCFA synthesis compared with people with migraine, which might be beneficial in maintaining their health [47]. *Bifidobacterium* preparation has been shown to relieve depression symptoms, and related products have been utilized as useful supplementary treatments in depression and anxiety [124]. 

In this regard, the role of the immune system in the GBA and in migraine pathobiology is supported by the data indicating that hypernociception generated by inflammatory stimuli can diminish in germ-free mice compared to normal mice [125]. Like migraine, inflammation is strongly implicated in depression/anxiety as well [126,127]. Indeed, individuals with depression tend to have greater baseline levels of circulating pro-inflammatory mediators, especially TNF-α and Interleukin-6 (IL-6) [128,129], compared to healthy individuals. In this context, it appears to be particularly relevant that *Ruminococcus gnavus*, which was increased in both depressive/anxiety [81] and migraine [47], was associated with intestinal diseases and exhibits pro-inflammatory properties by producing inflammatory polysaccharides, promoting gut pro-inflammatory responses and impairing gut barrier function [130]. Interestingly, *Bifidobacterium* was found to be reduced in migraine and depression [28,85]. It is known that it can exert anti-inflammatory effects, improve the permeability of the intestinal barrier and regulate the tryptophan levels and HPA axis [124]. Therefore, it has been found to be reduced in migraine and depression. Another relevant bacterium found to be decreased in both diseases [47,111] is *Faecalibacterium prausnitzii*, a butyrate-producing bacteria, which is crucial for intestinal health because it produces anti-inflammatory agents and aids in generating energy for colonocytes. Butyrate promotes cell proliferation and differentiation, increases the expression of BDNF, inhibits TNF-α, and affects 5-HT release [41]. BDNF is involved in the maintenance and survival of neurons as synaptic plasticity. Several lines of evidence involve BDNF in depression, as its expression is lower in depressed patients [131]. Several studies have shown that the exposure of exogenous corticosterone (to mimic the stress effect) also reduces BDNF expression in the rodent hippocampus, similar to that observed in various pre-clinical stress models [132,133]. Of note, many antidepressant treatments increase the levels of circulating BDNF [131,134]. 

Research supports the hypothesis that people with migraine show changes in the serotonergic system compared to healthy controls; notably, subjects with migraine have lower platelet 5-HT levels between attacks, which subsequently increases during a migraine attack [135]. In addition, deficits in the serotonergic system can result in various psychopathological conditions, including depression and anxiety [136,137]. *Faecalibacterium prausnitzii* components stimulate peripheral blood mononuclear cells, dendritic cells and macrophages to increase IL-10 production, thus inhibiting the pro-inflammatory cytokines like IL-6 and IL-12 [138]. The upregulation of inflammatory cytokines can have downstream effects in the brain, such as diverting tryptophan from 5-HT synthesis to the kynurenine pathway and excitotoxic/neurotoxic properties [139,140,141]. Indeed, the microorganisms in the gut can use tryptophan as a precursor for synthesizing a range of compounds such as kynurenine, quinolonate, indole, indole acetic acid, various indole derivatives, tryptamine and melatonin [139,142,143]. Some species of *Bifidobacteria* can produce neurotransmitters like acetylcholine and GABA [140,144]. Among SCFAs-producing bacteria, *Catenibacterium* has been found to be enriched in patients with both depression and migraine [49,94]. Although SCFAs have a beneficial role in both migraine and depression, they can exert pro-inflammatory effects in an amount- and time-dependent way, worsening the neuroinflammatory condition [145], suggesting that the interactions among bacteria in the gut environment are complex. The gut bacterial strain *Eggerthella* has been previously related to depression [146], and it is involved in the synthesis of 5-HT and GABA. *Eggerthella* levels were also consistently greater in patients suffering from anxiety and migraine [52] (Figure 1). Specifically, it was proposed that *Eggerthella lenta* may reduce dopamine levels [147]. This hypothesis by Rekdal et al. (2019) may explain the positive link discovered between *Eggerthella* and depressive levels [147] and probably also migraine associated with comorbidity. In addition, this bacterium induces gut inflammation by activating Th17 cells, confirming the dysbiosis relationship of the gut microbiota with depression and migraine [148].

Lastly, the pro-inflammatory genus *Clostridium* was found to be increased in cases of migraine and depression [47,78]. The genus includes many different species of *Clostridium* with a wide variety of functions; therefore, it is difficult to identify the underlying mechanisms by which it influences these conditions, and further studies are necessary. 

## 7. Potential Mechanisms Related to Microbiota and Relevant to the Drug Response in Migraine with Psychological Disorders

The comorbidity of migraine and depression/anxiety is strongly discussed. There are various potential causes, including one-way causal explanations, shared environmental and/or genetic risk factors and their interaction at multiple levels. By contrast, the bidirectional mechanisms explaining how the gut may induce depression/anxiety in migraine patients and vice versa are not entirely known.

Migraine attack symptoms are indeed positively correlated with the severity of psychopathologies [149,150] and appear to be more often unresponsive to migraine treatments incurring medication abuse [26,151,152,153]. 

The association between migraine and psychopathologies is strong, suggesting that it is unlikely to be coincidental. The gut microbiota are affected by various external factors, such as lifestyle and stress, which are common factors involved in migraine and psychological vulnerabilities, and this suggests an overlapping biological mechanism [154]. Stressful life events, or even psychological conditions, appear to occur before the switch from EM to CM [155,156,157]; indeed, a migraine symptoms burden seems to increase in a setting of stress, partially driven by psychiatric comorbidities [19]. Moreover, chronic stress, recognized as a risk factor for migraine and depression, has been linked to the dysregulation of the HPA axis, which manifests as higher cortisol levels and changes in the immune system and, specifically, in gut microbiota [158]. However, acute antimigraine treatments (e.g., nonsteroidal anti-inflammatory drugs (NSAIDs)) can affect the gut microbiota composition and metabolic activity through a direct effect on the microbiota or through an indirect effect by interacting with the host (e.g., changing the gut environment, mucosa integrity and permeability) [113]. In agreement, it was demonstrated that NSAIDs users can present different gut microbiota profiles from those of nonusers [159]. In turn, since many individuals with migraine suffer from overuse headache, it can be hypothesized that the persistent dysbiosis of the microbiota may influence drug metabolism and efficacy. Not only do therapeutic drugs affect gut microbes, but gut microbes can also metabolize drugs, affecting their pharmacodynamics, pharmacokinetics and toxicity [160]. Several studies have suggested two primary mechanisms for reciprocal interactions between medications and microbes: (1) biotransformation, which involves the chemical transformation of drugs by microbes, and (2) bioaccumulation, where bacteria store the drug intracellularly without modifying it chemically and without affecting their growth [161]. While biotransformation can be attributed to metabolic enzymes [162], drug bioaccumulation is a mechanism yet to be elucidated. However, bioaccumulation by gut bacteria is assumed to modulate the therapeutic effects of host-targeted drugs, with a primary action due to reduced drug availability and a secondary effect due to the secretion of modified metabolites [125]. In this context, it could be hypothesized that the identification of intestinal bacteria in people with migraine associated with psychopathologies may help to provide important pieces of information on the contribution of the alteration of the microbiota to the response to treatment.

**Figure 2:**
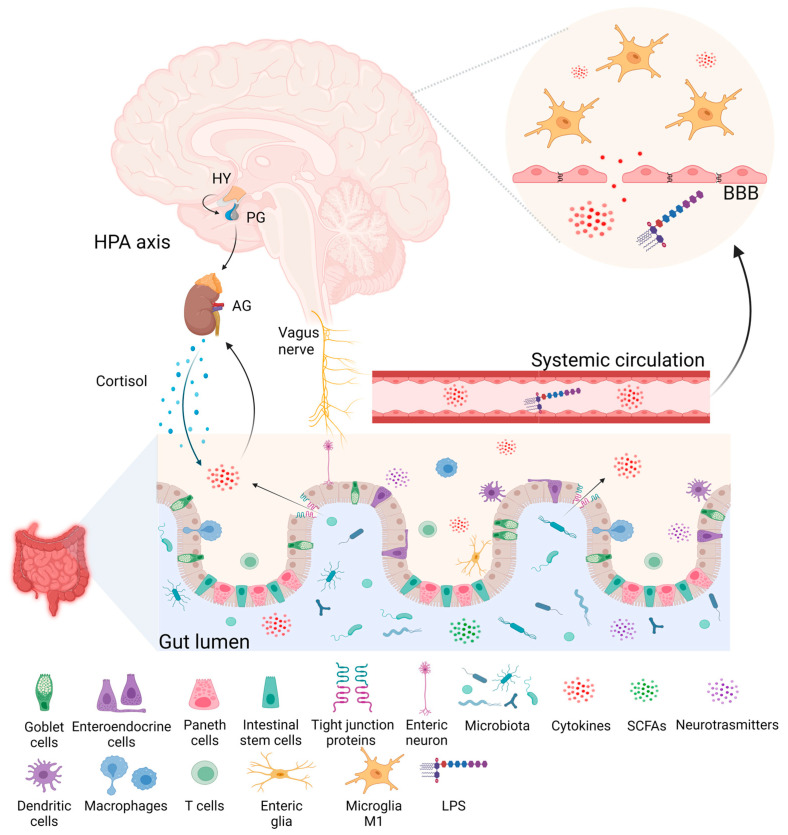
The gut microbiota can modulate the gut–brain axis and send signals to the central nervous system (CNS) through the activation of the vagus nerve and the interaction with the immune and endocrine systems. This latter also bidirectionally communicates with the gut microbiota, mainly via the hypothalamic-pituitary-adrenal (HPA) axis. In turn, the CNS can influence gut functions, intestinal permeability, the mucosal immune response and the composition of the microbiota. Physical or psychological chronic stress or pharmacological treatment can lead to an imbalance in gut bacteria and inflammatory factors, causing an increased permeability of the protective layer of the gut. Bacterial endotoxins, such as lipopolysaccharide (LPS), and cytokines leak from the gut lumen into the systemic circulation and trigger low-level inflammation. They can also travel to the brain and disrupt the blood–brain barrier (BBB) integrity by downregulating tight junction proteins, affecting adhesion proteins and the extracellular matrix. Bacteria-derived metabolites and LPS influence the development, maturation and function of the CNS resident immune cells, especially microglia, which adopts a proinflammatory phenotype and contributes to the development of neuroinflammation. Bacteria in the gut produce molecules like neurotransmitters and short-chain fatty acids (SCFAs) that can directly or indirectly stimulate brain receptors. This stimulation can trigger neural, endocrine and immune responses, potentially leading to depression, anxiety and migraine pain. PG: pituitary gland. AG: adrenal gland. Figure created with BioRender.com (https://www.biorender.com/ accessed on 11 June 2024).

In a clinical study, it has been observed that baseline differences in the microbiota may be correlated with antidepressant medication efficacy and thus contribute to the treatment response [163]. In addition, several studies have reported that the gut microbiota of depressive patients, responders or non-responders to treatments, exhibited significant differences in terms of both their microbial composition and metabolic pathways [163,164,165]. The genus *Bacteroides* is reduced in a treatment-nonresponsive depressed subgroup compared to in healthy controls [78], and it has a rich arsenal for metabolizing more complex carbohydrates, including glycans of human mucin [166,167]. In agreement with this hypothesis, Vuralli et al. [168] reported that an increased intestinal permeability, probably related to gut microbiota changes, would impact the response to anti-CGRP monoclonal antibodies in people with migraine. Figure 2 shows the microbiota–GBA crosstalk in migraine and depression.

## 8. Conclusions

Alterations in the gut microbial composition, in terms of microbial diversity and the relative abundance of different bacterial taxa, have been found in several neurological disorders, including migraine and psychopathology disorders. The gut microbiota of migraine patients and subjects with psychological disorders show a significant change in species diversity and metabolic functions but also an overlapping of common altered species, as extensively shown. The existing literature suggests that the maintenance of gut microecosystem stability improves headaches and associated symptoms, like depression and anxiety. Diets, dietary components or specific probiotics will probably help reduce the pathogenic microorganisms and restore dysbiosis in these patients. Thus, the identification of potential candidate bacterial taxa would inspire researchers to conduct further in-depth investigations in order to decode the pathways underlying migraine and comorbid conditions. 

The relationship between migraine, psychopathological disorders and changes in intestinal microbiota is a topic of growing research interest. While it is clear that these conditions often co-occur, establishing the direction of their relationship is complex. From one side, research in this field has shown a significant comorbidity between migraine and psychological disorders, including anxiety and depression, suggesting a bidirectional relationship where each condition may influence the other [169]. From the other side, the GBA seems to play a crucial role concerning the neurological and psychological state such that changes in the gut microbiota composition have been associated with both migraine and psychological disorders [144]. For instance, it has been shown that alterations in the gut microbiota can lead to or exacerbate conditions like depression, anxiety and migraine [170]. Conversely, psychological/physical stress and neurological conditions can alter the gut microbiota environment, leading to dysbiosis [171]. However, the most likely conclusion is a complex and bidirectional interaction where gut microbiota and the comorbidity of migraine and psychopathologies influence each other, creating a vicious cycle [172].

To date, the underlying mechanisms of observed relationships remain elusive and require further research on the relationship between preventive medications and gut microbiota, especially in CM. However, heterogeneity among study methods, such as variations in the sample collection, data analysis and sequencing procedures, results in a certain degree of complexity that limits the adequacy of information. Additionally, the diversity of the microbiota and its sensitivity to individual lifestyles, genetics and geographical locations make the generalization of data difficult.

## Figures and Tables

**Table 1:** Main bacteria altered in clinical and preclinical studies of migraine disease, with their products and the observed effects.

Bacteria	Alterations	Products (Metabolites, Amino Acids, Vitamins, Neurotransmitters)	Effects	References
*Agathobacter* (genus)	↑ ^c^		Negative association with severe headache intensity	[49]
*Alcaligenes* spp. (genus)	↑ ^c^			[52]
*Alistipes* (genus)	↑ ^p^	Indole and derivatives	Pro-inflammatory activity	[59]
*Akkermansia muciniphila* (species)	↓ ^p^	5-HT	Anti-migraine activity	[66]
*Bacteroides* (genus)	↑ ^p^	Tryptophan pathway metabolites, 5-HT, GABA, SCFAs		[63]
*Bifidobacterium* (genus)	↓ ^c^	Tryptophan, Folate, Pyroxidine, riboflavin, folate, niacin	Anti-migraine activity	[29]
*Bifidobacterium adolescentis* (species)	↓ ^c^	SCFAs (mainly acetate), GABA	Anti-inflammatory, antinociceptive activities	[47]
*Catenibacterium* (genus)	↑ ^c^	SCFAs		[29]
*Clostrium* spp. (genus)	↑ ^c^	Tryptophan	Pro-infalmmatoy activity	[47]
*Clostridium coccoides* (species)	↑ ^c^		Association with the severity of migraine symptoms	[52]
*Clostridium propionicum* (species)	↑ ^c^	Propionate		[52]
*Coprococcus* (genus)	↓ ^p^	SCFAs (mainly butyrate)	Anti-inflammatory activity	[63]
*Desulfovibrio* (genus)	↓ ^p^	Ammonia production, amino acid breakdown, 5-HT	Pro-inflammatory effects	[63]
*Eggerthella lenta* (species)	↑ ^c^	Arginine, dopamine modulation gut Th17 cells activation	Pro-nociceptive activity	[47,52]
*Eubacterium_g4* (genus)	↑ ^c^			[49]
*Faecalibacterium* (genus)	↓ ^c^	SCFAs (butyrate, D-lactate)	Anti-inflammatory activity	[47]
*Faecalibacterium prausnitzii* (species)	↓ ^c^	Butyrate	Anti-inflammatory activity	[47]
*Lachnospiraceae* (family)	↑ ^p^	SCFAs		[66]
*PAC000195_g* (genus)	↑ ^c^		Associated with a lower headache frequency	[49]
*Prevotella* (genus)	↑ ^p^	SCFAs (mainly butyrate, propionate)	Anti-inflammatory activity	[63]
*Rhodococcus* spp. (genus)	↑ ^c^			[52]
*Roseburia* (genus)	↑ ^c^	SCFAs (mainly butyrate)		[49]
*Ruminococcus* (genus)	↑ ^p^	Metabolizes tryptophan to tryptamine SCFAs, L-glutammate		[63]
*Ruminococcus gnavus* (species)	↑ ^c^	Intestinal mucin degradation SCFAs	Pro-inflammatory activity	[47]
*Streptococcus* (genus)	↑ ^p^	5-HT, dopamine, and norepinephrine		[63]
*Tissierellia* (classis)	↑ ^c^			[49]

↑, increased; ↓ decreased; ^c^, clinical studies; ^p^, preclinical studies.

**Table 2:** Main bacteria altered in clinical and preclinical studies of depression/anxiety disorders, with their products and the observed effects.

Bacteria	Alterations	Products (Metabolites, Amino Acids, Vitamins, Neurotransmitters)	Effects	References
*Alistipes* (genus)	↑ ^c^	Indole and derivatives	Pro-inflammatory activity	[78,86]
*Akkermansia muciniphila* (species)	↓ ^p^	5-HT, SCFAs	Modulation of the immune system, metabolic system, and endocannabinoid system	[109]
*Bacteroides* (genus)	↑ ^c^	Tryptophan pathway metabolites, 5-HT, GABA, SCFAs	Susceptibility to depression-like behavior	[82]
*Bifidobacterium* (genus)	↓ ^c^	Tryptophan, Folate, Pyroxidine, riboflavin, folate, niacin	Anti-depressive activity	[91]
*Catenibacterium* (genus)	↑ ^c^	SCFAs	Associated with depression severity	[94]
*Clostrium* spp. (genus)	↑ ^c^	Tryptophan	Pro-infalmmatoy activity	[78]
*Clostridium propionicum* (species)	↑ ^c^	Propionate		[93]
*Coprococcus* (genus)	↓ ^c^	SCFAs (mainly butyrate)	Anti-inflammatory activity	[84,87]
*Desulfovibrio* (genus)	↑ ^c^	Ammonia production, amino acid breakdown, 5-HT	Pro-inflammatory effects	[85]
*Dialister* (genus)	↓ ^c^	Butyrate	Anti-inflammatory activity	[79,83,87]
*Eggerthella* (genus)	↑ ^c^	5-HT, GABA		[83]
*Faecalibacterium* (genus)	↓ ^c^	SCFAs (butyrate, D-lactate)	Anti-inflammatory activity	[78,79]
*Faecalibacterium prausnitzii* (species)	↓ ^p^	Butyrate	Anti-inflammatory activity	[111]
*Lachnospiraceae* (family)	↑ ^c^/↓ ^c^	SCFAs		[78,112]
*Prevotellaceae* (family)	↓ ^c^	SCFAs	Anti-inflammatory activity	[83]
*Prevotella* (genus)	↓ ^c^	SCFAs (mainly butyrate, propionate)	Anti-inflammatory activity	[78,83]
*Roseburia* (genus)	↓ ^c^	SCFAs (mainly butyrate)	Anti-inflammatory activity	[77,80]
*Ruminococcus* (genus)	↓ ^p^/↓ ^c^	Metabolizes tryptophan to tryptamine SCFAs, L-glutammate		[78,108]
*Ruminococcus gnavus* (species)	↑ ^c^/↓ ^c^	intestinal mucin degradation SCFAs	Pro-inflammatory activity	[78,80,81]
*Sutterella* (genus)	↓ ^c^		Pro-inflammatory activity	[73,79]

↑, increased; ↓ decreased; ^c^, clinical studies; ^p^, preclinical studies.
